# The Crimping and Expanding Performance of Self-Expanding Polymeric Bioresorbable Stents: Experimental and Computational Investigation

**DOI:** 10.3390/ma11112184

**Published:** 2018-11-04

**Authors:** Fan Zhao, Laijun Liu, Yang Yang, Fujun Wang, Lu Wang

**Affiliations:** 1 College of Textiles, Donghua University, Shanghai 201620, China; zhaofan_dhu@163.com (F.Z.); liulaijunanhui@163.com (L.L.); 824811603@163.com (Y.Y.); 2 Key laboratory of Textile Science and Technology, Ministry of Education, College of Textiles, Donghua University, Songjiang District, Shanghai 201620, China

**Keywords:** composite polymeric bioresorbable stents, crimping and expanding, viscoelasticity, compression force

## Abstract

Polymeric bioresorbable stents (PBRSs) are considered the most promising devices to treat cardiovascular diseases. However, the mechanical weakness still hampers their application. In general, PBRSs are crimped into small sheathes and re-expanded to support narrowed vessels during angioplasty. Accordingly, one of the most significant requirements of PBRSs is to maintain mechanical efficacy after implantation. Although a little research has focused on commercial balloon-expanding PBRSs, a near-total lack has appeared on self-expanding PBRSs and their deformation mechanisms. In this work, self-expanding, composite polymeric bioresorbable stents (cPBRSs) incorporating poly(*p*-dioxanone) (PPDO) and polycaprolactone (PCL) yarns were produced and evaluated for their in vitro crimping and expanding potential. Furthermore, the polymer time-reliable viscoelastic effects of the structural and mechanical behavior of the cPBRSs were analyzed using computational simulations. Our results showed that the crimping process inevitably decreased the mechanical resistance of the cPBRSs, but that this could be offset by balloon dilatation. Moreover, deformation mechanisms at the yarn level were discussed, and yarns bonded in the crossings showed more viscous behavior; this property might help cPBRSs to maintain their structural integrity during implantation.

## 1. Introduction

Polymeric bioresorbable stents (PBRSs), which can maintain luminal patency even when entirely absorbed after vascular remodeling [1,2], are the subject of broad research for cardiovascular diseases. Unfortunately, studies show that acute stent recoil occurs a lot in PBRSs, leading to in-stent restenosis [3]. These undesirable features are mainly caused by insufficient mechanical efficacy under consistent external loads [4,5]. Accordingly, the mechanical stability of PBRSs remains the principle obstacle to their safe use.

However, a considerable amount of research on the mechanical characteristics of PBRSs focuses on the improvement of radial compression through the use of ideal materials and structures, while ignoring stent behaviors that occur during angioplasty operating processes [6]. Actually, stents experience crimping and expanding loads when implanted [7]. Large deformations happen in stent struts. For balloon-expanding polymer stents, plastic deformations occur because of struts strains that are higher than the yield points of material stress-strain curves. This might negatively affect the durability of PBRSs. Although stents may show outstanding radial resistance in vitro, they can experience high mechanical loss under the implantation process. Thus, except for radial resistance, another significant performance aspect of PBRSs is their ability to withstand deformations due to being crimped into smaller sheathes, and being expanded at the lesion, while maintaining comparative mechanical efficacy to open the artery. Crimping processes cause extreme anisotropy in polymeric stents [8], as well as transformations in the polymer chain orientation from hoop direction to the radial direction on a micrometer scale. This may lead to stent ductility [9,10]. Moreover, polymers have lower stiffness than alloy, and greater strains are generated during crimping and expanding. Thus, dog-boning and recoiling happen in polymeric stents [11]. This can introduce residual stresses in stents, and cause further degradation [12,13,14,15]. Schiavone et al. reported lower expansion rates and higher recoil of the Absorb scaffold (Abbott Vascular, Santa Clara, CA, USA), compared with Xience V stents [16]. Thus, mechanical assessment of stents, especially polymeric ones, without considering the effect of crimping and expanding processes, may fall short in terms of accuracy. All these factors suggest that stent deformations under crimping and expanding are significant, and must be factored into stent design and life-prediction.

Unlike metallic stents, there is little literature about PBRS deployment processes, although a few studies refer to commercialized balloon-expanding devices [16,17,18]. However, these devices rely on significant plastic deformation to resist recoiling and keep the blocked artery open, which can introduce severe residual stress in the stents [7]. This stress may increase to the ultimate tensile strength of the material, leading eventually to strut fracture [10]. Nevertheless, self-expanding polymer stents can self-oppose to the vessel wall. The elastic deformation contributes to their shrinking during the crimping process, and can be recovered during expanding [17]. However, analysis of their crimping and expanding deformation mechanisms is lacking in the literature.

The main objective of this study was to further mechanical research of self-expanding PBRSs under crimping and expanding processes, as well as to elucidate the relationship between their design and stability. In our former study, we developed composite self-expanding stents that incorporate poly(*p*-dioxanone) (PPDO) monofilaments and Poly(ε-caprolactone) (PCL) multifilaments based on braiding technology [19], which are aimed at congenital heart disease. We provided an experimental method for crimping and expanding evaluation of polymer prototypes in vitro. According to this methodology, mechanical stability evaluations were conducted on self-made cPBRSs. The deformation mechanisms that occurred during the crimping and expanding process were examined by numerical simulation. The results not only advance our understanding of braid deformation mechanisms during the angioplasty process, but also point to a route for optimizing PBRS mechanical stability.

## 2. Materials and Methods

### 2.1. Materials

Poly(*p*-dioxanone) (PPDO) monofilaments and Polycaprolactone (PCL) multifilament were manufactured by melt spinning in Donghua University (Shanghai, China). Composite braided yarns (cBYs) were made by wrapping the core PPDO monofilament with four groups of PCL multifilament on an 8-bobbin braiding machine. Composite melted yarns (cMYs) were made by thermal-treating cBYs at 90 °C for 1 h in air. According to the American Society for Testing and Materials Standard (ASTM) D2256-2010, breaking strengths and breaking elongation rates of raw materials and two types of yarns were tested by Darong Universal Mechanical Tester (Model YG-B026H, Darong Textile Instrument Co., Ltd., Wenzhou, China). The initial gauge length was 250 mm, with a test velocity of 500 mm/min. The physical parameters of yarns are shown in Table 1. In addition, cPBRSs were formed by PPDO monofilaments and cMYs, and the mechanical performance of stents was supported by yarn mechanical deformations. Thus, the stress-strain curves of PPDO monofilaments and cMYs were also measured to help us to explore cPBRS deformation mechanisms during the crimping and expanding process.

### 2.2. Fabrication of Composite Polymeric Bioresorbable Stents (cPBRSs)

**Figure 1:**
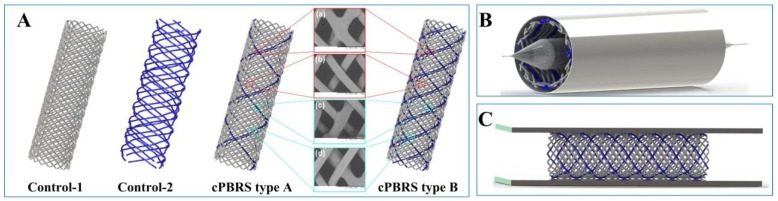
Schematic diagram of PBRSs crimping and expanding measurement. (**A**) Schematic diagram and morphology of different prototypes; (**B**) Crimping and balloon-expanding measurement; (**C**) Parallel plate measurement. (Grey yarns represent PPDO monofilaments, dark blue yarns represent cMYs).

A 32-bobbin braiding machine was used to fabricate prototype composite stents [19] in the Biomedical Textile Materials Research Laboratory of Donghua University, Shanghai, China. The cBYs were braided with PPDO monofilaments to form composite polymeric bioresorbable stents (cPBRSs). Notably, prototype stents with four groups of the cBYs were defined as cPBRS type A, while those with eight groups of the cBYs were termed cPBRS type B (Figure 1A). Since two components contribute to cPBRSs, their roles were considered separately by using two control prototypes: prototypes braided by 32 PPDO monofilaments (the Control-1 prototype) and 8 cBYs (the Control-2 prototype), respectively. All of them were braided onto a cylindrical mold with an outer diameter of 8 mm to guarantee uniform inner diameters and the same braiding angle. Afterward, all samples were thermally treated in air for 1 h in an electrothermal blowing dryer (Shanghai Yiheng Co., Ltd, Shanghai, China) at 90 °C, which is higher than the melting point of the PCL multifilament. 

### 2.3. Crimping and Expanding Performance

Crimping and expanding performance was evaluated to simulate the process in which stents were compressed into sheaths during implantation and then released after reaching the site. A radial load was applied by a crimper to radially compress the stents from a nominal diameter to 3.8 mm. After holding for 300 s, the crimper unloaded the stents gradually. Afterward, samples were divided into two groups. For one group, samples were balloon-expanded immediately after the crimping process, aiming at mitigating polymer viscoelasticity influence. A 10 Fr balloon was used with 4 atm to expand 3 times and 30 s each time (Figure 1B) [20], while another group of samples recovered from deformations by self-expansion. 

In order to explore the effects of the crimping process on the morphology of stents, especially the viscous response of the polymer, the diameters of prototypes was measured at three stages: before crimping, after self-expanding immediately, and after self-expanding for 24 h. The effects of balloon-dilatation on stent morphology were also evaluated after stent balloon-expansion immediately as well as 24 h after this process. Parallel plate measurement was applied to evaluate stent compression performance after the crimping and expanding process, according to the International Organization for Standardization (ISO) 25539-2012. A customized compression resistance instrument (Model LLY-06D, Laizhou Electronic Instrument Co., Ltd., Laizhou, China) was used (Figure 1C). Prototypes were compressed to 50% deformation of outer diameter, with the maximum load recorded and defined as the compression force [21]. Considering that time-reliable recovery occurred, samples were tested after self-expansion or balloon-dilatation for 24 h in order to explore the mechanical loss of different prototypes after the crimping and expanding process.

### 2.4. Numerical Analysis

#### 2.4.1. Material Models

##### Viscoelastic Material Modeling

Significant viscoelasticity was observed in semi-crystallized polymeric stents during the crimping test. Thus, hereditary integrals were adopted to build linear viscoelastic material models.

The constitutive equations of shear creep compliance *J* and shear modulus *G* for a linear viscoelastic material were given by the hereditary integral and reported by Shanahan et al. [22]:

(1) $$\varepsilon \left(t\right)=\tau_{0}J\left(t\right)+\int_{0}^{t}J\left(t-s\right)\dot{\tau }\left(s\right)ds$$

(2) $$\tau \left(t\right)=\gamma_{0}J\left(t\right)+\int_{0}^{t}G\left(t-s\right)\dot{\gamma }\left(s\right)ds$$

where τ(t) is the time-dependent shear stress and *G*(*t*) is the time-dependent shear modulus.

The shear relaxation modulus *G*(*t*) can be derived as the Prony series accordingly, and subsequently expressed by normalized shear relaxation modulus g(t) for Abaqus input:

(3) $$G\left(t\right)=G_{0}-\overset{N}{\underset{i=1}{\sum }}G_{i}\left[1-e^{\left(-t/\tau_{i}\right)}\right]$$

(4) $$g\left(t\right)=1-\overset{N}{\underset{i=1}{\sum }}g_{i}\left[1-e^{\left(-t/\tau_{i}\right)}\right]$$

where *N*, *g*(*i*), and $\tau_{i}$ are material constant.

Normalized shear creeps compliance data were required to fit the Prony series constitutive equation. Considering the tensile creep test, which was easier to conduct, we measured PPDO monofilaments and cMYs with concentrated force of 5 N in the Darong Universal Mechanical Tester (Model YG-B026H, Darong Textile Instrument Co., Ltd., Wenzhou, China) for 18000 s. Afterward, data were further converted to shear compliance by the following equation. Here, we assumed them to be homogeneous isotropic.

(5) $$J\left(t\right)=\frac{2\left(1+v\right)}{E}=2\left(1+v\right)\frac{\varepsilon \left(t\right)}{\sigma_{0}}$$

The conversion of creep data was reported in Table A1 of Appendix A.

After that, the nonlinear least-squares fit of the creep data to the Prony series was executed to determine the coefficients and the relaxation periods. In addition, the time-dependent normalized shear relaxation modulus curves of PPDO monofilaments and cMYs were obtained in Equations (6) and (7).

(6) $$g\left(t_{P}\right)=1-\left\{0.12212\left[1-e^{\left(-t/163.53\right)}\right]+6.24168E-02\left[1-e^{\left(-t/3438.6\right)}\right]\right\}$$

(7) $$g\left(t_{c}\right)=1-\left\{0.13789\left[1-e^{\left(-t/149.01\right)}\right]+9.64757E-02\left[1-e^{\left(-t/2862.2\right)}\right]\right\}$$

where $g\left(t_{P}\right)$ and $g\left(t_{c}\right)$ are the time-dependent normalized shear relaxation modulus of PPDO monofilaments and cMYs used in this study, respectively.

##### Validation of the Material Models

**Figure 2:**
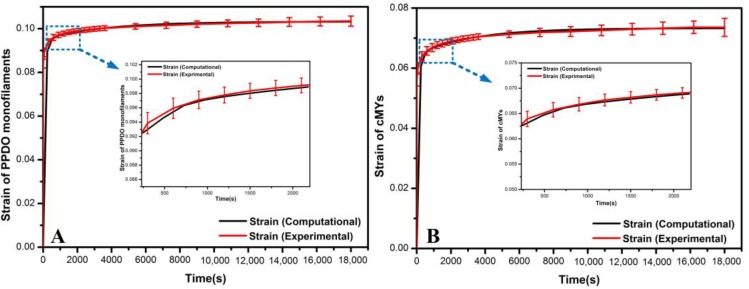
Comparison between the tensile creep tests obtained experimentally and the Abaqus predictions: (**A**) PPDO monofilaments; (**B**) cMYs.

The linear viscoelastic material models of PPDO monofilaments and cMYs were validated by “Visco” step in Abaqus/Explicit. Two 3D deformable parts were built and assigned with creep data of PPDO monofilaments and cMYs, respectively, which were calculated in part 2.4.1.1 and reported in Appendix A. Displacement/Rotation boundary conditions were applied to one end of the parts by constraining all movements. A concentrated force of 5 N was applied to the other end, and lasted 18000 s. Afterward, simulated yarn tensile strain-time curves were compared to those of the tensile creep test, and showed good agreement, as shown in Figure 2. These results validated the viscoelastic material models for the PPDO monofilaments and cMYs used in this study.

#### 2.4.2. Finite Analysis Procedure

Finite element analysis was used to simulate the crimping and balloon expanding processes of the stents. Abaqus/Explicit was used as the solver to easily achieve convergence. The input parameters include geometrical models, material models. The stent prototype models, crimping tool, and balloon were built with Solidworks software (Version 2012, SolidWorks Corp., Dassault Systemes, Concord, MA, USA). The four stent models were defined by length (30 mm), inner diameter (8 mm), fiber diameter (0.30 mm), number of fibers (32), and braiding angle (55°). The original crimping tool and balloon were defined by a length (50 mm and 30 mm, respectively) and diameter (9.2 mm and 8 mm, respectively). All models were assumed to be isotropic entities. cMY and PPDO monofilament were modeled with viscoelastic properties [23]. Linear elastic models were adopted for the crimping tool and balloon to simplify the calculations. The finite element mesh of the considered prosthesis was created automatically by HyperMesh (version 14.0) and imported into Abaqus (version 6.14, Dassault Systèmes Simulia Corp., Providence, RI, USA) subsequently. 8-node hexahedral elements with full integration (C3D8) and 8-node hexahedral elements with reduced integration (C3D8R) element were used for meshing polymer yarns and a crimping tool. Also, 4-node shell elements with reduced integration (S4R) were conducted for the balloon [18]. The approximate global sizes were set at 0.1 and 1 mm respectively. Hard contact formulation and penalty function with a friction coefficient of 0.25 was assigned for the contact surfaces. The displacement condition was used to simulate the crimping process by decreasing the diameter of the crimping tool from 9.2 mm to 3.8 mm. Then, the balloon expansion process was executed by increasing the balloon’s outer diameter from 8 mm to 10 mm. Afterward, stress and strain distributions of prototypes were presented to analyze the difference of PPDO monofilaments and cMYs during the crimping and balloon-expansion simulation, which constituted to the cPBRSs. Moreover, viscoelastic deformations of semi-crystallized polymers can consume and dissipate energy, and cause prototype mechanical loss during unloading. Thus, the distributions of creep dissipation energy on different yarns were also analyzed.

### 2.5. Statistical Analysis

T-tests were used for determining the statistical difference between samples. The data reported was the means and standard deviations, and the error bars in the figures corresponded to standard deviations. The data in the figures were marked by * for *p* < 0.05.

## 3. Results

### 3.1. Stress-Strain Curves of PPDO Monofilaments and cMYs

**Figure 3:**
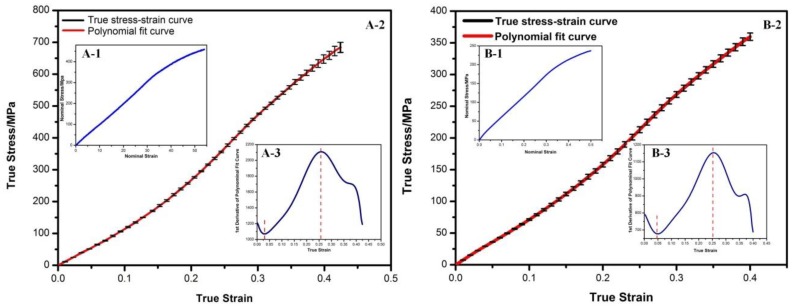
Mechanical properties of PPDO monofilaments and cMYs. (**A**) PPDO monofilaments: (**A-1**) Measured stress-strain curves; (**A-2**) Calculated stress-strain curves, (**A-3**) 1st derivative of the polynomial fit curve. (**B**) cMYs: (**B-1**) Measured stress-strain curves; (**B-2**) Calculated stress-strain curves, (**B-3**) 1st derivative of the polynomial fit curve.

Measured stress-strain curves in uniaxial tensile test (Figure 3(A-1,B-1)) are commonly used to evaluate yarn mechanical properties. However, it is inappropriate to represent the real elastic modulus changes, since yarn diameters alter continuously during tensile deformation. Thus, some formulations were used to calculate the real yarn stress-strain curve transformed from experimental uniaxial tensile data [24].

(8) $$\varepsilon_{true}=\int_{l_{0}}^{l}\left(\frac{dl}{l}\right)=\ln\left(1+\varepsilon_{nom}\right)$$

(9) $$\sigma_{true}=\frac{F}{A}=\sigma_{nom}\left(1+\varepsilon_{nom}\right)$$

where *A* is the cross area of the yarn in the tensile process, *l* is the yarn length in the tensile process.

Accordingly, the calculated stress-strain curves of PPDO monofilaments and cMYs are shown in Figure 3(A-2,B-2), respectively. In order to analyze their slope changes, polynomial fits were used in software Origin 8.5 to match the calculated curves. Both of them showed high agreements (R^2^ = 0.999 and 0.995, respectively). Accordingly, the first derivative was further calculated. Similar trends with three zones were observed for PPDO monofilaments and cMYs. For PPDO monofilaments, the slope declined in the first 3.0% strain of calculated stress-strain curve, followed by the reinforced zone, since the modulus increased continuously up to 26.0%. A fracture occurred with hardening behavior, which was not obvious (Figure 3(A-3)). For cMYs, reinforced zone laid in 5.2% to 26.3% (Figure 3(B-3)).

### 3.2. Crimping and Expanding Performance

**Figure 4:**
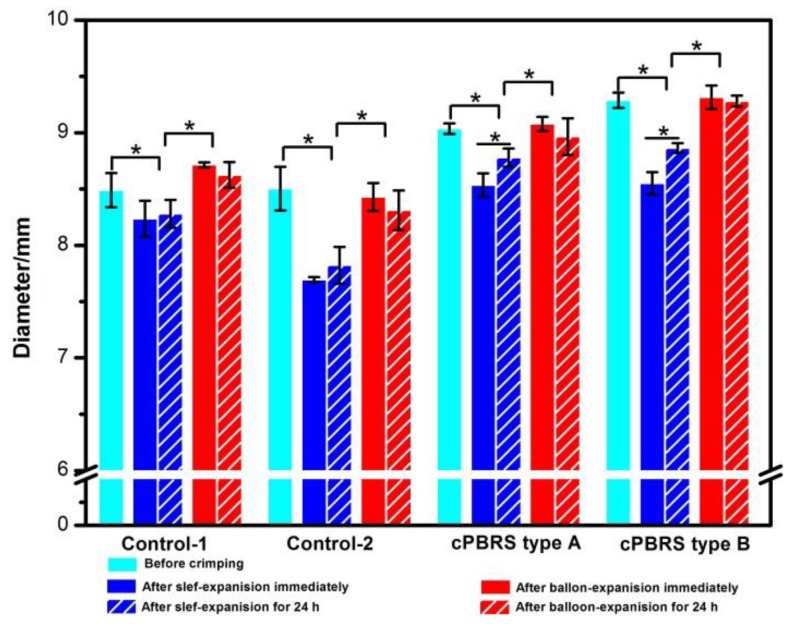
Diameter changes of different prototypes during crimping and expanding process. (* means *p* < 0.05).

The results of prototype outer diameters after the crimping and expanding process are shown in Figure 4. Regarding the effect of the crimping process on the cPBBS type A and type B, stent diameter decreased by 5.53% and 7.93% after immediately self-expanding. The values recovered by 2.85% and 3.66% after time-reliable recovery, respectively, compared with those tested immediately after the crimping process. The values of the Control-1 and the Control-2 showed similar trends to the cPBRSs. Moreover, stent diameters can be recovered to origins for all prototypes immediately after balloon-expanding. The diameters of the Control-1 after balloon-dilatation were even higher than their original values mainly because of structural failure.

**Figure 5:**
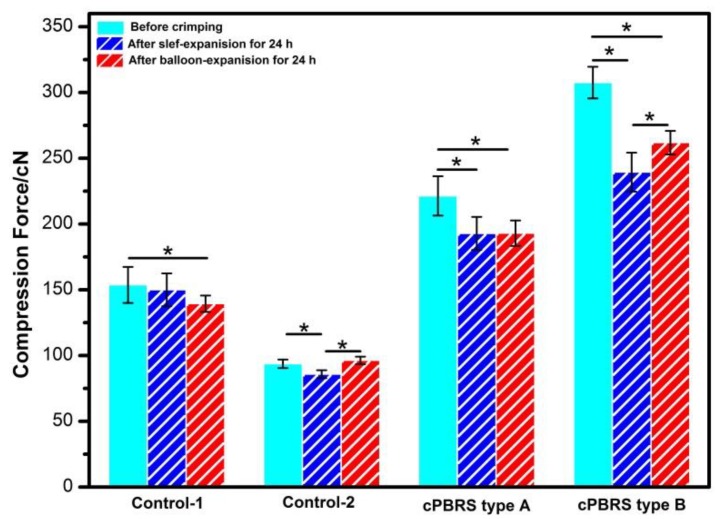
Compression force changes of different prototypes during crimping and expanding process. (* means *p* < 0.05)

Figure 5 illustrates the compression force after crimping and expanding process. With regards to cPBRSs, the crimping process decreased the mechanical force significantly, by 12.90% for the cPBBS type A and 22.14% for the cPBRS type B. The same trend was observed in the Control-2. However, there was a slight decrease in the Control-1 without statistical difference. The compression force recovery rates of the cPBRS type B and the Control-2 were significantly higher after balloon expansion compared with self-expansion. Specifically, the Control-2 nearly recovered to its original value after balloon-dilation. However, the result was the opposite for the Control-1, with the values decreasing by 7.23% after balloon-induced expansion compared to self-expanding.

### 3.3. Computational Results

#### 3.3.1. Crimping Simulation Results

**Figure 6:**
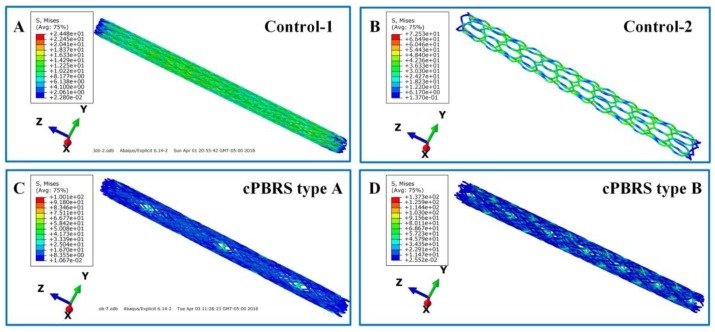
The Von Mise distributions of different prototypes after crimping to 3.8 mm in diameter. (**A**) The Control-1 prototype; (**B**) The Control-2 prototype; (**C**) The cPBRS type A prototype; (**D**) The cPBRS type B prototype.

The Von Mise stress distributions of the prototypes after crimping are shown in Figure 6. Stents elongated to fit the diameter changes and the profiles remained uninformed. The stress distributed evenly in the body of the Contro-1, with lower stress in both ends (Figure 6A). For the Control-2 prototype, the stress concentrated in the zones around bonding points, and it led to more bending yarns (Figure 6B). For cPBRSs, higher stress was concentrated on the cMYs, as shown in Figure 6C and Figure 6D. In addition, the values of the stress increased with the number of the cMYs.

**Figure 7:**
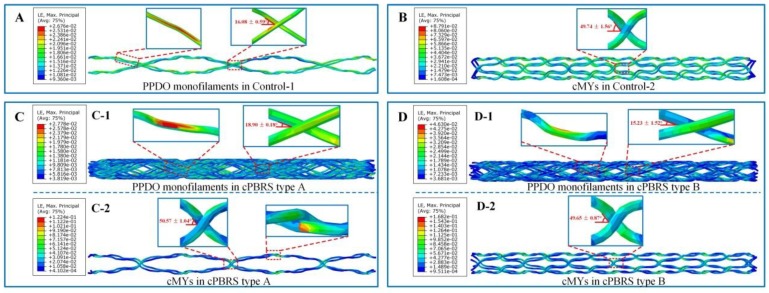
The logarithmic strain distributions of typical yarns in different prototypes after crimping simulation. (**A**) Typical PPDO monofilaments of the Control-1. (**B**) cMYs of the Control-2. (**C**) Different components of the cPBRS type A: (**C-1**) PPDO monofilaments; (**C-2**) cMYs. (**D**) Different components of the cPBRS type B: (**D-1**) PPDO monofilaments; (**D-2**) cMY. (Braiding angles of all prototypes before crimping process were 54.21 ± 0.60°).

Figure 7 illustrates the logarithmic strain distributions of typical yarns in different prototypes after the crimping process. As shown in Figure 7A, the braiding angles of the Control-1 prototype decreased dramatically, with the strain distributed evenly along with the longitudinal direction of stents. The cMYs of Control-2 (Figure 7B) exhibited higher strain (8.79% in maximum) compared with yarns in the Control-1, which was mainly concentrated around the bonding points. As for cPBRSs, the strain distributions of different components behaved significantly differently. In particular, PPDO monofilaments for the cPBRS type A (Figure 7(C-1)) behaved the same as those in the Control-1, while more serious bending was observed for the cPBRS type B (Figure 7(D-1)). The cMYs for both cPBRS type A (Figure 7(C-2)) and type B (Figure 7(D-2)) behaved larger deformations than that in the Control-2. Specifically, the maximum strain of the cMYs for the cPBRS type A prototype reached 12.4% and increased to 16.8% for the cPBRS type B prototype.

**Figure 8:**
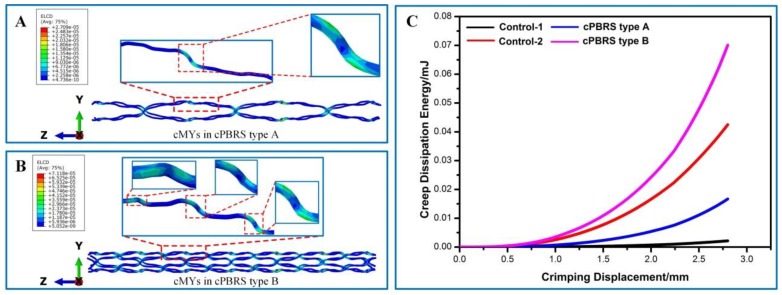
Creep dissipation energy of different prototypes during crimping simulation. (**A**) Creep dissipation energy distributions of cMYs in cPBRS type A prototype; (**B**) Creep dissipation energy distribution of cMYs in cPBRS type B prototype; (**C**) Crimp dissipation energy–displacement curves of different prototypes.

Considering semi-crystallized polymers used in this study (PPDO and PCL), viscous behavior inevitably occurred during yarns bending. Creep dissipation energy was used to explore uneven deformations for cPBRSs, as shown in Figure 8. Higher bending deformations observed in cMYs of cPBRSs than other prototypes can contribute to significant viscous behavior. Thus, this phenomenon of the cMYs for cPBRSs was analyzed specifically. As shown in Figure 8A,B, the creep dissipation energy was mainly concentrated around the bonding points. The values increased with the number of the cMYs. As shown in Figure 8C, the total amount of creep dissipation energy increased significantly with crimping displacement. In addition, PPDO monofilaments of the cPBRS type B were speculated to show viscoelasticity, since the values were higher than those of the Control-2 prototype.

#### 3.3.2. Balloon Expanding Simulation Results

**Figure 9:**
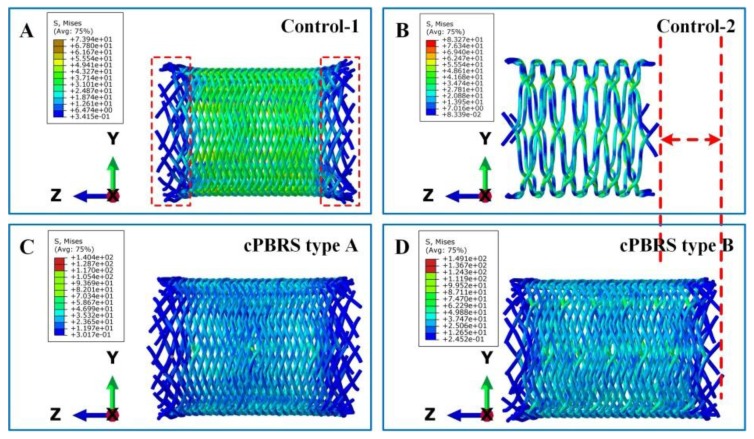
The Von Mise distribution of different prototypes after balloon-expanding simulations. (**A**) The Control-1 prototype; (**B**) The Control-2 prototype; (**C**) The cPBRS type A prototype; (**D**) The cPBRS type B prototype.

Figure 9 illustrates the Von Mise stress distributions of different prototypes after balloon expansion. For the Contro-1 (Figure 9A), stress distributed evenly on the body of stents, while dog-bone shape with a higher diameter in both ends was observed. The stress concentrated around the bonding points for the Control-2 (Figure 9B) and the profile remained uniform and stable. Moreover, cPBRSs also showed a uniformed profile and larger stresses concentrated on the cMYs, especially for the cPBRS type B (Figure 9C,D). In addition, the stress values increased with the cMYs number as intensified by the interaction between cMYs and PPDO monofilaments.

**Figure 10:**
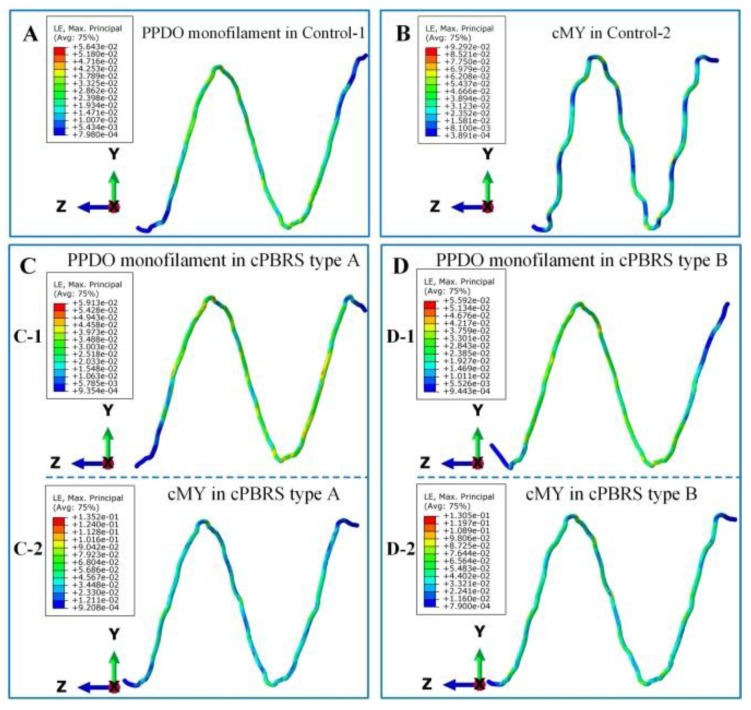
The logarithmic strain distributions of typical yarn in different prototypes after balloon-expanding simulation. (**A**) PPDO monofilament of the Control-1 prototype. (**B**) cMY of the Control-2 prototype. (**C**) Different components of the cPBRS type A prototype: (**C-1**) PPDO monofilament; (**C-2**) cMY. (**D**) Different components of the cPBRS type B prototype: (**D-1**) PPDO monofilament; (**D-2**) cMY.

Since all prototypes deformed symmetrically during balloon-expansion, typical yarns of different prototypes were analyzed specifically, as shown in Figure 10. As for the PPDO monofilaments, the strains spread evenly and different prototypes showed similar values, with the maximum higher than 5.5% (Figure 10(A,C-1,D-1)). Larger deformations were observed in cMYs of all prototypes compared with PPDO monofilaments. In particular, composite melt yarn (cMY) for the Control-2 prototype arranged closely and tightly with the maximum strain as high as 9.29% (Figure 10B). Moreover, the values of cMYs in cPBRSs (Figure 10(C-2,D-2)) were higher than 16.0% in maximum, mainly derived from the interaction between different components.

**Figure 11:**
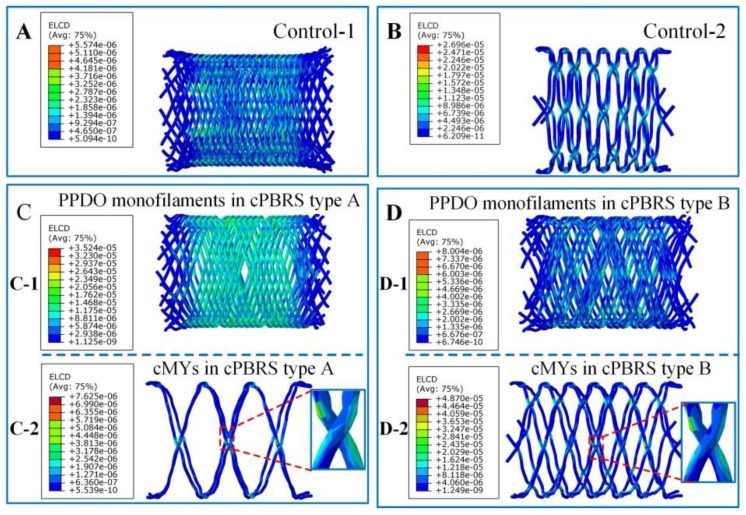
Creep dissipation energy distributions of different prototypes after the balloon-expanding simulations. (**A**) The Control-1 prototype. (**B**) The Control-2 prototype. (**C**) Different components of the cBPRS type A prototype: (**C-1**) The PPDO monofilaments; (**C-2**) The cMYs. (**D**) Different components of the cBPRS type B prototype: (**D-1**) PPDO monofilaments; (**D-2**) cMYs.

As shown in Figure 11, all stents behaved viscoelasticity to some extent. Lower creep dissipation energy was observed on PPDO monofilaments for the Control-1 (Figure 11A), the cPBRS type A (Figure 11(C-1)) and the cPBRS type B (Figure 11(D-1)), with the maximum values almost ten times lower than that of the cMYs. Higher creep dissipation energy distributed on cMYs for cPBRSs, compared with the Control-2, indicating the viscoelasticity of cMYs were intensified by the PPDO monofilaments. Moreover, this phenomenon was more severe with the increasing number of cMYs as shown in Figure 11(C-2,D-2). 

## 4. Discussion

The ability to maintain stent mechanical performance after deployment during angioplasty is one of the critical issues for PBRSs [25]. However, little attention has been paid to this factor, especially when deformation mechanisms are involved. We have previously developed composite bioresorbable stents (cPBRSs) which have promoted the mechanical properties of polymeric stents significantly. However, due to their viscoelastic properties [22,23], polymeric prototypes show the drawback of time-reliable recovery after implantation. Stent recovery to original diameters and sufficient mechanical forces after being released from sheath are crucial for stent fixation under blood flow [26,27]. A balloon has been used to accelerate stent expansion. In this study, we explored the mechanical behavior of cPBRSs during the crimping process. The influence of balloon dilatation influence on the prototype mechanical properties was also evaluated. The experimental results showed that the mechanical forces decreased after the crimping process, while being offset by balloon dilatation. Moreover, different roles played by PPDO monofilaments and cMYs in the cPBRSs mechanical maintenance were discussed by the combination of material calculated stress-strain curves and computational simulations.

In the crimping and expanding process, two deformation modes were involved for cPBRSs: PPDO monofilaments rotation in the crossings and cMYs bending. Different deformations can affect the behavior of polymer chains and result in various physical and mechanical properties of yarns. For PPDO monofilaments and cMYs used in this study, most polymer chain segments were unable to move before 3% and 5.2% yarn strain, respectively (Figure 3). Thus, only elastic deformation of yarns occurred, which can be recovered quickly and entirely. While more external work, chain segments were removable and aligned with the external load. Time-reliable changes of chain conformation happened accordingly (3% to 26% yarn strain for PPDO, 5.2% to 26.3% yarn strain for cMYs), and deformed yarns demonstrated viscoelasticity (Figure 3). In particular, energy was consumed in order to break existed secondary bonds and overcome internal friction between different chain segments to stimulate conformation changes [28]. As a result, plastic deformation occurred after unloading [29]. The mechanical properties of yarns reduced to some extent during reloading, while it strengthened when loading in opposite direction.

Besides, different deformation modes of PPDO monofilaments and cMYs can further affect the mechanical properties of cBRPSs. They were considered separately by analyzing the results of the Control-1 and the Control-2. The Control-1 prototype was formed by PPDO monofilaments, with 2.68% yarns strain in maximum after crimping process (Figure 7). Little creep dissipation energy was observed. Accordingly, the mechanical force did not change significantly under radial loading. However, the structural failure of both ends highly contributed to the mechanical loss during the balloon-expanding process (Figure 5). For cMYs, formed the Control-2 prototype, the viscous deformation occurred significantly during strains higher than 5% in both crimping and balloon-expanding process (Figure 7 and Figure 10). Thus, the mechanical loss of the Control-2 prototype occurred after the crimping process, while it recovered entirely through balloon dilatation (Figure 5).

Moreover, the results of this study indicated that the interaction between PPDO yarns and cMYs also played an important role in affecting the physical and mechanical characteristics of cPBRSs during crimping and expanding process. Specifically, skeleton formed by cMYs can keep the structure stable, and did not behave dog-boning during balloon-expansion as the Control-1 did (Figure 9). More stress concentration zones observed on PPDO monofilaments and cMYs for cPBRSs, resulted in more inhomogeneous stress distribution when compared with the Control-1 and the Control-2. Thus, higher yarn bending degrees occurred in these areas for cPBRSs in order to deform to confined dimension after crimping process. In this case, more severe viscous behaviors were obtained and led to more mechanical loss during crimping process (Figure 5). In addition, only cMYs behaved higher strain and more serious viscoelasticity than the Control-2, while PPDO monofilaments remained elastic deformation the same as the Control-1 (Figure 10). Hence, the mechanical loss of cPBRSs cannot be recovered entirely by balloon dilatation (Figure 5). 

In this work, specific holding time and residual stresses generated in the crimping and expanding process were not considered. This will involve a significant amount of additional work and will be considered in our future studies. However, it does not affect the general conclusion of this paper, as we aimed to analyze the deformation mechanisms of different components of the cPBRSs during crimping and expanding process. 

## 5. Conclusions

We have experimentally simulated the implantation process on the cPBRSs by the self-developed device. Besides, the computational simulation revealed the deformation mechanisms of different prototypes during this process. Viscoelasticity was verified occurred mainly in the cMYs during crimping process while happened in both PPDO monofilaments and the cMYs during the balloon-expanding process. This leads to the variety of dimensional and mechanical changes for different prototypes accordingly. Moreover, the mechanical loss during the crimping process can be recovered by balloon dilatation and better effects were obtained with more cMYs in the cPBRSs. The results of this study may open new prospects for the mechanical evaluation of the polymeric bioresorbable stents and might inspire future development of advanced devices.

## Figures and Tables

**Table 1:** Physical parameters of yarns.

Type of Yarns	Diameter/mm	Breaking Strength/MPa	Breaking Elongation/%	Melting Temperature/°C
PPDO monofilaments	0.30 ± 0.01	433.17 ± 21.71	53.50 ± 1.09	102.73 ± 0.15
PCL multifilament	0.14 ± 0.02	199.02 ± 0.01	47.64 ± 0.03	56.03 ± 0.21
cBYs	0.48 ± 0.01	182.72 ± 15.92	55.61 ± 3.70	-
cMYs	0.43 ± 0.01	221.49 ± 4.01	61.58 ± 6.20	-

## Appendix A

**Table A1:** Normalized shear creep compliance data converted from tensile creep test.

Time (s)	Normalized Shear Creep Compliance *j*(*t*) ± SD	
	PPDO Monofilaments	cMYs
1	1.00 ± 0.024	1.00 ± 0.036
60	1.06 ± 0.023	1.07 ± 0.029
300	1.12 ± 0.018	1.14 ± 0.027
600	1.14 ± 0.017	1.17 ± 0.025
900	1.16 ± 0.015	1.19 ± 0.024
1200	1.16 ± 0.013	1.21 ± 0.021
1500	1.17 ± 0.012	1.22 ± 0.020
1800	1.18 ± 0.013	1.23 ± 0.019
2100	1.18 ± 0.012	1.23 ± 0.018
2400	1.18 ± 0.013	1.24 ± 0.016
2700	1.19 ± 0.013	1.24 ± 0.017
3000	1.19 ± 0.013	1.25 ± 0.016
3300	1.19 ± 0.012	1.25 ± 0.016
3600	1.19 ± 0.013	1.26 ± 0.017
5400	1.20 ± 0.015	1.27 ± 0.019
7200	1.21 ± 0.017	1.28 ± 0.024
9000	1.21 ± 0.017	1.29 ± 0.029
10800	1.22 ± 0.019	1.30 ± 0.031
12600	1.22 ± 0.020	1.30 ± 0.034
14400	1.23 ± 0.024	1.31 ± 0.037
16200	1.23 ± 0.025	1.31 ± 0.037
18000	1.23 ± 0.028	1.31 ± 0.053
